# Epileptic seizures in multiple sclerosis: prevalence, competing causes and diagnostic accuracy

**DOI:** 10.1007/s00415-020-10346-z

**Published:** 2020-12-15

**Authors:** Friederike Neuß, Felix von Podewils, Zhong Irene Wang, Marie Süße, Uwe Klaus Zettl, Matthias Grothe

**Affiliations:** 1 Department of Neurology, University Medicine Greifswald, Ferdinand-Sauerbruchstraße, 17475 Greifswald, Germany; 2 Epilepsy Center, Neurological Institute, Cleveland Clinic Foundation, Cleveland, OH USA; 3 Department of Neurology, Neuroimmunological Section, University of Rostock, Rostock, Germany

**Keywords:** Multiple sclerosis, Epilepsy, Seizures, Comorbidity, Epidemiology

## Abstract

**Background:**

Multiple sclerosis (MS) is accompanied by an increased risk of epileptic seizures, but data with a detailed description of the competing causes are lacking.

**Methods:**

We aimed to describe a cohort of patients with both MS and epileptic seizures in a retrospective, population-based study.

**Results:**

We included 59 out of 2285 MS patients who had at least one epileptic seizure. Out of them, 22 had seizures before the diagnosis of MS, whereas epileptic seizures occurred after MS diagnosis in 37 patients, resulting in a total prevalence of epileptic seizures in MS of 2.6%. Competing causes could be found in 50.8% (30/59) of all patients, with 40.9% (9/22) compared to 56.8% (21/37) of the MS patients with seizures before vs after MS diagnosis. The main alternative causes were traumatic brain injury and cerebral ischemia accounting for more than 30% of the patients, with no difference between the subgroups. 33.3% and 55.6% of MS patients with seizures before/after MS diagnosis had documented pathological EEG alterations.

**Conclusion:**

A remarkable percentage of MS patients with epileptic seizures do have alternative competing causes at the time of the first seizure. A detailed diagnostic setup including patient history, EEG and MRI is recommended in the evaluation and choice for the best treatment.

## Introduction

The association between multiple sclerosis (MS) and epileptic seizures has been described for more than 30 years [1]. Several recent studies confirmed a threefold increased risk for epileptic seizures in MS patients compared to healthy controls [2–5]. Seizures can occur as first symptom in MS [6–8], but the cumulative incidence rises with disease duration up to nearly 6% [2].

The underlying cause of epileptic seizures in MS is not well understood. Imaging studies in small samples of well characterized MS patients suggest grey matter pathology particularly in the temporal lobes to be associated with a higher risk of epilepsy [9–11].

On the other hand, seizures can be caused by several other brain pathologies such as traumatic injury, infection, neoplasia, or stroke [12–14], which can also occur in MS, especially with increasing disease duration [15]. In a very recent study, the rate of epilepsy in MS was much lower after excluding all alternative etiologies, suggesting that the causes of epilepsy in MS might be heterogeneous [16].

Another important issue is the diagnostic accuracy. Detailed information on how the diagnosis of epilepsy was initially made is often not available, especially in registry studies. According to the guidelines of the International League Against Epilepsy (ILAE), diagnosis of epilepsy can be made after two unprovoked seizures occurring more than 24 h apart, after one unprovoked seizure with a high recurrence risk or after diagnosis of an epilepsy syndrome [17, 18]. Apart from clinical appearance of seizures, electroencephalogram (EEG), especially in combination with provocation methods, is seen as an important tool to differentiate between epileptic and non-epileptic seizures [19]. Existing data suggest that up to 70% of patients with epilepsy are misdiagnosed, highlighting the importance of a detailed clinical assessment and EEG in the diagnostic process [20].

Taken together, the literature about epilepsy in MS is rather insufficient. The aim of this retrospective study, therefore, was to characterize a cohort of MS patients with known epileptic seizures more precisely with respect to competing risk factors, onset of seizures in relation to MS onset, as well as diagnostic procedures and accuracy. To investigate the effect of comorbidities on the relationship between epilepsy and MS, we further dichotomized the cohort in two subgroups, one with MS diagnosed after the onset of seizures (S-MS) and another with seizures occurring after the diagnosis of MS (MS-S).

## Materials and methods

The retrospective population-based cohort study was approved by the local ethics committee of the University medicine Greifswald (BB022/19). We screened medical reports from the MS outpatient clinics from the departments of Neurology, University medicine Greifswald and University medicine Rostock, for patients who have been treated in at least one of these two specialized centers between 03/2009 and 03/2019. Both hospitals offer specialized MS treatment in the entire catchment area of about 750,000 people.

Clinical data were collected by reviewing the patients’ medical records and included age, gender, disease course of both MS as well as epileptic seizures and/or epilepsy, including progression in time, treatment patterns and severity. Comorbidities were classified in categories according to ICD-10 codes [21]. Patients were excluded if medical reports were incomplete, patients did not fulfill diagnostic criteria for MS [22, 23], or the characterization of seizures was questionable. All variables were collected at the time of the first seizure as well as at the time of the last reported visit.

SPSS 21 (IBM Co., Armonk, New York, USA) was used for statistical processing of the data. For the group statistics for the competing causes, Fishers exact test was computed with the significance level set at *p* < 0.05.

## Results

### Selection of cohort

**Fig. 1:**
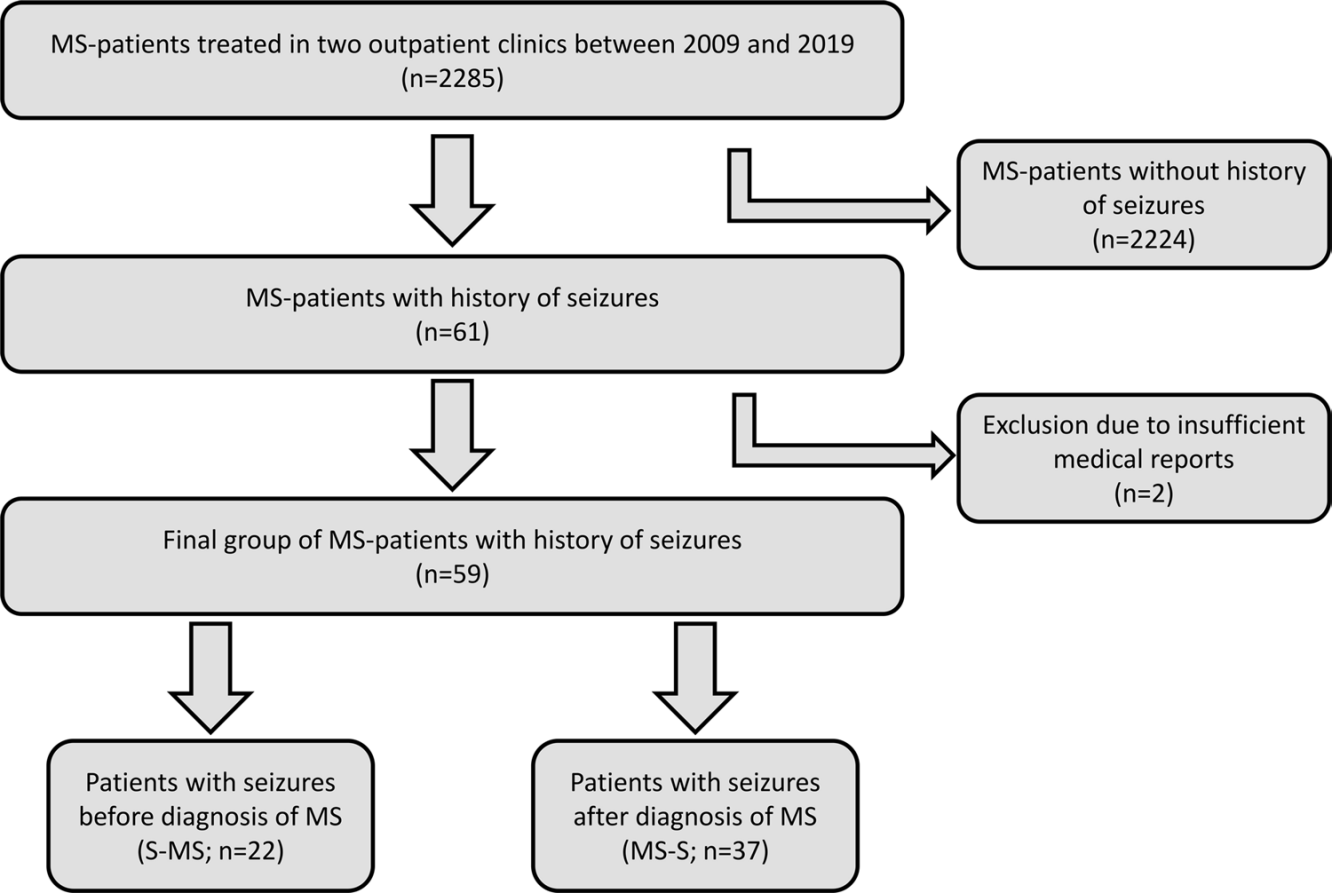
Flowchart summarizing the selection of patients that constitute our final cohort

Between 2009 and 2019, a total of 2285 MS patients were treated in the two university outpatient clinics in Rostock and Greifswald. Out of these, a group of 61 MS patients was defined with at least one epileptic seizure. Two patients were excluded due to insufficient documentation. Therefore, our final cohort consists of 59 patients, resulting in a prevalence of 2.6% (Fig. 1).

### Demographical and clinical data

Female patients consisted of 76.3% (45/59) of all patients. Mean age at last reported visit was 51.7 years (SD 13.6), median EDSS 5.0 (QR 2.0–6.5). At time of last reported visit, 52.5% (31/59) had relapsing–remitting disease course (RRMS), 42.4% (25/59) had secondary-progressive MS (SPMS) and 5.1% (3/59) primary-progressive MS (PPMS) (see Table 1).

**Table 1:** Demographical and clinical data

	All MS patients (*n* = 59)	S-MS (*n* = 22, 37.3%)	MS-S (*n* = 37, 62.7%)
Age (years/mean)	51.7	46.2	55.0
EDSS (median)	5.0	3.0	6.0
Disease duration at time of last reported visit (month/mean)	181.6	98.3	232.5
Disease course at time of last reported visit (*n*, % of patients in group)			
RRMS	31, 52.5	17, 77.3	14, 37.8
SPMS	25, 42.4	4, 18.2	21, 56.8
PPMS	3, 5.1	1, 4.5	2, 5.4

The most frequently used anti-seizure drugs (ASD) at last reported visit was levetiracetam (32.2%, 19/59), lamotrigin (25.4%, 15/59), valproate (10.2%, 6/59), and carbamazepin (8.5%, 5/59). Nine patients out of 59 (15%) were treated with a combination of two ASD, two (3%) with a combination of three ASD (see Table 2), whereas 27.1% (16/59) of patients had no ASD.

**Table 2:** Antiepileptic treatment at time of last visit (patients with more than one medication; ^a^in two patients; ^b^in one patient each)

Patients with double-therapy			
Lamotrigine	Lacosamide		^a^
Lamotrigine	Mesuximide		^b^
Lamotrigine	Valproate		^b^
Levetiracetam	Clobazame		^b^
Levetiracetam	Lacosamide		^b^
Levetiracetam	Oxcarbazepine		^b^
Oxcarbazepine	Gabapentin		^b^
Valproate	Carbamazepine		^b^
Patients with triple-therapy			
Lamotrigine	Levetiracetam	Eslicarbazepinacetate	^b^
Lamotrigine	Levetiracetam	Lacosamide	^b^

According to the available medical reports, 41.8% (23/55) of the MS patients only had one single epileptic seizure. Of those with recurrent seizures 32.7% (18/55) had not more than one seizure per year and 25.5% (14/55) had one or more seizures per year.

At the time of the first seizure, 26.8% (15/56) of the patients received disease-modifying-treatment (DMT) for MS, consisting of interferon-beta (14.3%, 8/56), glatiramer acetat (3.6%, 2/56), dimethylfumarat (1.8%, 1/56), fingolimod (1.8%, 1/56), mitoxantron (1.8%, 1/56), natalizumab (1.8%, 1/56), methotrexat (1.8%, 1/56). Three patients had to be excluded for this analysis due to missing information on the DMT.

At the time of the last documented visit, pharmacological treatment of MS consisted of fingolimod (15.3%, 9/59), interferon-beta (13.6%, 8/59), glatiramer acetat (6.8%, 4/59), dimethylfumarat (3.4%, 2/59), mitoxantron (1.7%, 1/59) or natalizumab (1.7%, 1/59). 57.6% (34/59) of the patients had no disease modifying therapy on last recorded visit.

#### Diagnostic accuracy of epilepsy

Due to the retrospective design and documentary weaknesses, no consistent information on the epilepsy classification is available.

Approximately half of the epileptic seizures were witnessed by neurologists (14.0%, 8/57) or other physicians (28.1%, 16/57), but the majority of the seizures (57.9%, 33/57) were only documented according to observations from non-medical professionals. Two patients had to be excluded for this analysis due to missing information about seizure documentation.

Information about EEG could be obtained in 48 out of 59 patients, which always was routine EEG. In 58.3% (28/48) of these patients, pathological EEG alterations (regional or generalized slowing, focal or generalized epileptiform discharges) were documented, whereas 41.7% (20/48) had normal EEG findings.

In total, one-third of the seizures (33.3%, 16/48) were diagnosed and classified only according to patient documentation (i.e. without pathological EEG and without direct observation).

#### Competing causes of epilepsy

In our cohort of 59 patients, 30 (50.8%) had risk factors documented in the medical reports that can cause epileptic seizures and can therefore be an alternative cause, such as traumatic brain injury (18.6%, 11/59), cerebral ischemia (10.2%, 6/59), migraine (*n* = 5, 8.5%), brain tumor (*n* = 3, 5.1%), and drug abuse (*n *= 3, 5.1%) (details in Table 3).

**Table 3:** Competing causes for seizures and p-values for group comparison

Risk factor	Total (*n*, % of patients)	MS-S (*n*, % of patients)	S-MS (*n*, % of patients)	*p*
*n* =	59	37	22	
None	29, 49.2%	16, 43.2%	13, 59.1%	0.29
Traumatic brain injury	12, 20.3%	7, 18.9%	5, 22.7%	0.48
Cerebral ischemia	6, 10.2%	4, 10.8%	2, 9.1%	1
Migraine	5, 8.5%	3, 8.1%	2, 9.1%	1
Brain tumor	3, 5.1%	3, 8.1%	0, 0%	0.54
Drug abuse	3, 5.1%	3, 8.1%	0, 0%	0.54
Intracranial hemorrhage	2, 3.4%	2, 5.4%	0, 0%	0.53
Infantile brain damage	2, 3.4%	1, 2.7%	1, 4.5%	1
Meningioma	2, 3.4%	2, 5.4%	0, 0%	0.53
Meningitis	2, 3.4%	2, 5.4%	0, 0%	0.53
Psychological trauma	2, 3.4%	1, 2.7%	1, 4.5%	1
Alcohol abuse	1, 1.7%	1, 2.7%	0, 0%	1
Brain abscess	1, 1.7%	1, 2.7%	0, 0%	1
Developmental venous anomalies	1, 1.7%	0, 0%	1, 4.5%	0.36
Encephalitis	1, 1.7%	0, 0%	1, 4.5%	0.36
Epidural hematoma	1, 1.7%	1, 2.7%	0, 0%	1
Sinus vein thrombosis	1, 1.7%	0, 0%	1, 4.5%	0.36
Stereotactic biopsy	1, 1.7%	1, 2.7%	0, 0%	1
Stereotactic deep brain stimulation	1, 1.7%	1, 2.7%	0, 0%	1
Subdural hematoma	1, 1.7%	0, 0%	1, 4.5%	0.36
Subdural hygroma	1, 1.7%	1, 2.7%	0, 0%	1

#### Group comparison S-MS vs. MS-S

A total of 37 patients (62.7%) showed seizures after the diagnosis of MS and were categorized into the MS-S subgroup; 22 patients (37.3%) showed seizures before MS was diagnosed and were categorized into the S-MS subgroup.

In the MS-S subgroup, clinical course at the time of the first epileptic seizure was RRMS in 16/37 (43.2%), SPMS in 19/37 (51.4%) and PPMS in 2/37 (5.4%).

Clinical course at the last recorded visit in MS-S was SPMS (56.8%, 21/37), RRMS in 14 patients (37.8%) and PPMS in 2 patients (5.4%); in S-MS RRMS (17 patients, 77.3%), SPMS in 4 patients (18.2%) and PPMS in one patient (4.5%).

#### Diagnostic accuracy of epilepsy between S-MS and MS-*S*

In S-MS, 55.6% of patients (10/18) had documented pathological EEG alterations. In contrast, in MS-S only 33.3% (10/30) had EEG alterations.

Diagnosis of epilepsy was made only according to patient documentation in 28.6% (6/21) S-MS patients and in 11 out of 36 (30.6%) patients of MS-S.

#### Competing causes of epilepsy between S-MS and MS-S

Although not significant (*p *> 0.25), patients with MS-S more frequently showed at least one additional risk factor for epileptic seizures in their patient history (21/37 patients; 56.8%) compared to those with S-MS, where only 40.9% (9/22 patients) showed at least one competing cause. The most frequent risk factors were traumatic brain injury (MS-S: 18.9% vs. S-MS: 22.7%), cerebral infarction (10.8% vs. 9.1%), migraine (8.1% vs. 9.1%), brain tumor (8.1% vs. 0%) and drug abuse (8.1% vs. 0%). Table 3 shows a complete list of competing causes.

## Discussion

The goal of this retrospective population-based study was to investigate in detail a cohort of MS patients with epileptic seizures or the diagnosis of epilepsy to better characterize patients’ demography, diagnostic accuracy, the onset of seizures in relation to MS onset as well as competing risk factors. For this purpose, we retrospectively analyzed medical reports of two large university outpatient clinics specialized in MS from the past 10 years. To our knowledge, this is the first study to systematically investigate MS and epileptic seizures in a population-based approach.

### Prevalence

We showed the prevalence of epileptic seizures in MS is 2.6%, which is in line with existing literature [2, 4] and higher compared to the general population [24]. Interestingly, in a recent study, the prevalence of epilepsy in MS was 0.9% using rather strict diagnostic criteria excluding patients with alternative etiologies [16]. Our data are also in line with this prevalence, as only 1.3% of all MS patients of our cohort had epileptic seizures with strict diagnostic criteria without any competing causes.

### Demographic and disease related characteristics

The mean age in our cohort was 51.7 years at the last visit, predominantly women, which is comparable to existing cohort and registry studies [2, 25]. A systematic review by Gasparini et al. [26] revealed no difference between relapsing and progressive courses, whereas Burman and Zelano showed a risk increase of epilepsy by the factor 2.5 [2] in progressive disease courses. This seeming contradiction is in line with our findings that MS patients with epilepsy were equally distributed between relapsing and progressive disease forms, but differed between the subgroups S-MS and MS-S. In our cohort, 37.3% had the first seizure before the diagnosis of MS was made. Of this subgroup, 77.3% of patients had a relapsing course at the time of the last visit. On the other hand, the most frequent course at the first epileptic seizure in MS-S was SPMS (51.4%), with 56.8% on the last reported visit, emphasizing the association with increasing disease duration in this subgroup. Additionally, we here describe a cohort of MS patients with seizures. As the vast majority (75%) of our patients treated in the outpatient clinics were relapsing remitting, the ratio is much higher in progressive patients as well.

In our cohort, a high proportion of more than 50% of the patients with MS did not have any disease modifying drug at the last documented visit, which could essentially be due to the high percentage of secondary-progressive MS patients. Because the choice of treatment depends on the year and the country of the data collection, study comparison is difficult. In a Swedish registry study in 2017, almost 15% of the MS patients did not have any MS treatment. However, the lower proportion of patients with SPMS in this registry [2] might explain the difference from our data. Time of data collection, national guidelines, time of drug approval as well as local preferences can also explain differences in the choice of ASD. In our cohort, levetiracetam and lamotrigin were the most frequently used ASD in almost half of our patients, whereas in a Norwegian study the majority of patients were treated with carbamazepine [25]. In total, antiepileptic treatment was found in 72.9% of our patients, which is comparable to existing data from MS registries [27].

### Diagnostic accuracy

To our knowledge, diagnostic accuracy of epileptic seizures and epilepsy in MS has not been investigated yet. According to the ILAE guidelines, diagnosis of epilepsy can be made after two unprovoked seizures occurring more than 24 h apart [17]. However, in MS, the ILAE guidelines may be more difficult to interpret in cases of only one unprovoked seizure, as the recurrence risk mainly depends on the detection of an MRI lesion responsible for the seizure [17]. Although the diagnosis of MS largely depends on the evidence of characteristic lesions in the cerebral MRI, there is a diagnostic uncertainty whether these lesions actually are relevant to a seizure onset zone, especially as cortical lesions are difficult to detect in routine imaging [28]. According to the ILAE classification, structural etiology “…refers to abnormalities visible on structural neuroimaging where the electro-clinical assessment together with the imaging findings lead to a reasonable inference that the imaging abnormality is the likely cause of the patient’s seizure…” [18]. It can be assumed that in a considerable proportion of MS patients, the diagnosis of structural epilepsy was perhaps made prematurely, since this prerequisite is difficult to prove.

Interestingly, the interictal EEG was pathologic in one-third of the MS-S subgroup and more than 50% in the S-MS subgroup. In the existing case series, pathological EEG was highly prevalent (13 of 13 patients in [25], 12 of 14 patients in [24]), but the inclusion criteria for these studies were different compared to the current study with our population-based approach.

Nevertheless, in one-third of our patients, epileptic seizures or even epilepsy was diagnosed only on history. Taken this into account and considering the very high rate of pathologic EEG findings in MS patients shown here, a detailed and well thought-out diagnostic scheme including EEG diagnostic is strongly recommended to further strengthen the diagnosis of epilepsy in MS and to exclude competing causes for seizures.

### Onset epilepsy in relation to MS

In 22 of 59 (37.3%) patients with MS and epilepsy, the first epileptic seizure occurred before the diagnosis of MS was made. Compared to other studies [6], this large proportion in our cohort was rather surprising. An association between the epileptic seizures and the later diagnosed MS cannot be clearly proven. However, none of these patients was diagnosed with epilepsy syndromes such as genetic generalized epilepsy. On the other hand, possible competing risk factors for epileptic seizures were found in nearly 40% of the patients in the subgroup of S-MS patients, most frequent traumatic brain injury. Taken this into account, one can hypothesize that early subtle and clinically irrelevant cortical lesions in the later manifest MS might already have caused the seizures.

There is a long debate if epileptic seizures might be the initial clinical manifestation of MS, and the existing literature (summarized and discussed in [29]) as well as our cohort study suggest that seizures can occur before the diagnosis MS is made. Some case series also suggest seizures occurring predominantly during relapses, or as the only clinical sign of a relapse [30]. In our cohort, we did not find any of such cases, although we cannot rule out the possibility of incomplete medical reports.

As this retrospective study only analyses existing medical reports, we did not have detailed information about the diagnostic that was made during the time the seizure occurred. Further prospective studies have to systematically investigate MS-related pathology like temporal cortical lesions that might lead to epilepsy in MS [10, 11].

### Competing risk factors

Alternative causes for epileptic seizures were highly frequent, seen in half of our cohort with a vast majority of structural etiologies. In several other studies, patients with competing risk factors have been excluded from further analysis [6, 16]. In a very recent study on epilepsy in multiple sclerosis, Langenbruch et al. excluded 16 out of 90 patients for further analysis because of other potential risk factors for epilepsy besides MS [16]. In a French cohort, 35 out of 102 patients with MS and epilepsy had documented alternative risk factors, mainly associated with not only early onset of the seizures in infancy (24 out of 35) [6], but also cerebral haemorrhage or tumor. In contrast, we aimed to document all potential competing risk factors for epilepsy in MS patients, which theoretically explains half the prevalence of our cohort. We are aware of the different epileptogenic risk of different underlying conditions; for example, brain tumor or intracranial hemorrhage are associated with a higher risk to cause epileptic seizures compared to migraine [31]. However, the basic idea of this study was to describe the risk of epileptic seizures and epilepsy in MS in a real-life approach.

Seizures always can be provoked by various pathologies other than MS such as traumatic injury, infection, neoplasia or stroke. According to a prospective study from Iceland, 9% of unprovoked seizures in adults are attributable to brain trauma, 7% to cerebrovascular disease [14], a Swedish epilepsy registry describes brain tumor (ca. 4%) and stroke (ca. 5%) as the main presumed etiologies of first seizures [32]. In our study, trauma (20%) and cerebral ischemia (10%) had been the main alternative causes as well, and brain tumor also identifiable in 5% of our patients. As these competing risk factors are descriptive, no further etiological allocation is possible.

Interestingly, no difference was seen with respect to risk factors between S-MS and MS-S. This can be interpreted as a systemically proportion of other CSN pathology leading to seizures. On the other hand, it might also be a hint that seizures may be part of MS, as there is no systematic difference in alternative causes.

### Limitations

Since our research has been carried out retrospectively and is based on patient documentation, in some cases the information collected was different, incomplete or in some cases missing altogether. For example, the type of epileptic seizures was rarely documented, which is why we dispensed with the evaluation completely. Additionally, the retrospective design and the inclusion of patients treated at specialized tertiary referral centers might be a source of a selection bias. Despite these limitations, this population-based approach for the first time ever gives a rather detailed overview about MS patients with epilepsy.

## Conclusion

Seizures in patients with MS are more frequent than in general population, although common assumptions on prevalence rates might be overestimated. Our real-life data on MS patients with epileptic seizures show that competing risk factors can be found in up to 50% of the patients and are comparable to other patients with structural epilepsy. However, the decision whether to start ASD treatment or not is largely dependent on the estimation of the risk of seizure recurrence. Therefore, to prove the association between seizures and MS and to exclude other etiologies as underlying causes, it is important to have detailed information about competing risk factors and to perform a targeted diagnostic including EEG and MRI.
